# Development and content validation of a questionnaire to assess the social determinants of mental health in clinical practice

**DOI:** 10.3389/fpsyt.2024.1377751

**Published:** 2024-05-20

**Authors:** Fritz Handerer, Peter Kinderman, Imogen Nevard, Sara Tai

**Affiliations:** ^1^ Division of Psychology and Mental Health, University of Manchester, Manchester, United Kingdom; ^2^ Institute of Primary Care & Mental Health, University of Liverpool, Liverpool, United Kingdom; ^3^ Division of Nursing, Midwifery and Social Work, University of Manchester, Manchester, United Kingdom

**Keywords:** screening, whole person approach, social risk assessment, clinical questionnaire development, social determinants of health

## Abstract

**Introduction:**

There is growing consensus that consideration of the Social Determinants of Mental Health should be at the centre of mental health care provision. To facilitate this, a validated means to assess mental health service users' social contextual information is arguably needed. We therefore developed a questionnaire to assess the Social Determinants of Mental Health in clinical practice.

**Methods:**

Our guideline-informed development consisted of three steps; i) construct and purpose definition, ii) initial item generation based on the literature, similar questionnaires, and a selection of the ICD-10, iii) evaluation, revision, and content validation of the questionnaire. Initially we developed 249 items that were reduced, revised, and validated in several stages to 73 items. Content validation of the questionnaire was achieved through surveys and focus groups including mental health care service users and professionals.

**Results:**

The surveys and focus groups indicated the need for a standardised assessment of adverse social factors and highlighted that the benefits of such an assessment would be a more holistic approach to identifying and addressing fundamental factors involved in the development of mental health difficulties. Importantly, this study also revealed how any assessment of the Social Determinants of Mental Health must prioritise the assessed person having a central role in the process and control over their own data. The focus groups identified contradicting recommendations regarding the most suitable context to administer the questionnaire.

**Discussion:**

The resulting questionnaire can be considered to be theoretically robust and partially validated. Future research is discussed.

## 1 Introduction

There is consensus that our mental health depends on the conditions we live in and the events that happen to us (1). It has been argued that those Social Determinants of Mental Health (SDMentH) are the main drivers of our health outcomes (2). Consequently, the Social Determinants of Mental Health should feature prominently in clinical practice (3). Analyses of medical records, however, revealed that social contextual information is rarely ever noted in English mental health care services (4), implying that SDMentH play only a minor role in current practice (5).

This inadequate consideration of SDMentH is partly due to an insufficient medical nomenclature. There are 853 codes in common medical coding systems (SNOMED, LOINC, ICD-10) to record social contextual information, which is too extensive to streamline observations (6). Furthermore, some of the most important social determinants, such as migration status, or structural racism are not addressed in current coding systems (7).

In order to standardise assessment of social contextual information on these grounds and to elevate the consideration of social contextual factors in clinical practice, assessment tools for the social determinants are needed. Such assessment tools serve several purposes. In the short term they inform the treatment an individual receives (8, 9), by identifying risk and protective factors (10), making treatment more holistic (11) and initiating referrals (12). Over the long term, a tool facilitated assessment of social contextual information could inform aetiological models (8) and policy making (13), increase health equality (14), and ameliorate collaboration between services (15). Early empirical findings indicate that applying assessment tools for social determinants in clinical practice leads to a more person-centred doctor patient interaction, improved care experiences, elevated population health, and decreased costs (16). Especially efficient are computer-based assessment tools, which are time economic and shown to have the highest disclosure rate (10, 17).

Within the last decade, numerous screening tools for the Social Determinants of (physical) Health (SDH) have been developed, mostly in the United States (18). These tools are intended for use in family medicine, oncology, emergency department, and gynaecology. No tool has been developed specifically for use in mental health care services. This is problematic, as a recent review of the literature conducted by this research team, revealed that the two constructs Social Determinants of (physical) Health and Social Determinants of Mental Health differ on epistemological and aetiological grounds, despite considerable overlap regarding the factors that are considered to be important (19). While the Social Determinants of (physical) Health are typically understood to affect health outcomes directly based on a positivist worldview, the Social Determinants of Mental Health are thought to affect mental health outcomes through indirect mechanisms, filtered through individual perspectives. It would therefore be theoretically inappropriate to utilise an assessment tool for the Social Determinants of physical Health in a mental health care setting. To address this gap, we developed a questionnaire to assess the Social Determinants of Mental Health in clinical practice to elevate and standardise the consideration of social contextual factors in mental health care.

## 2 Method

### 2.1 Study design

Development and validation of the questionnaire was guided by Artino and colleagues’ standard for questionnaire development (20) and is in line with Christalle’s best practice example for generating patient reported measures from qualitative data (21). The process comprised of three components; (i) the clear construct definition and determination of the questionnaire’s purposes, followed by (ii) an initial item generation, which were then (iii) evaluated, revised, and validated in a third step. The study received ethics approval by the University of Manchester UREC on the 20^th^ of July 2021, Ref: 2021-11448-20076.

**Table 1** provides an overview of the study design and the stages of development.

**Table 1:** Overview of study design.

Stage	Aim	Method/data source	Result
Preparation			
Preparation	Define construct and context of measurement	Scoping review of the literature on SDMentH	Definition as in section 1.1
Item generation			
Initial item generation	Create an exhaustive pool of items pertaining to the SDMentH	-Special Z-section of ICD -Existing SDH assessment tools -Domains as revealed by the scoping review	249 items
First revision of the pool of items	Remove duplicates and format the items in line with the construct definition	-Discussions within the research team including 2 clinical psychologists	98 items
Item selection, revision and validation			
First evaluation of content validity	Feedback from experts and members of the target population on content validity, acceptability, and completeness	Online survey for quantitative validation including free writing columns for comments (4 mental health care service users and 4 mental health care professionals)	• 14 items removed • 72 items reworded • 1 item added
Evaluation of content validity, acceptability, and application context	Feedback from experts and members of the target population on clarity, acceptability, and validity	3 focus groups (4 providers, 3 providers, 7 users) on zoom	• 16 items removed • 21 items reworded • 2 newly introduced item types
Second evaluation of content validity	Feedback from experts and members of the target population on content validity, acceptability, and completeness	Online survey for quantitative validation including free writing columns for comments (4 mental health care service users and 4 mental health care professionals)	• 2 items removed • 2 items merged with other existing items • 49 items reworded • 0 items added 73 items in the final questionnaire

#### 2.1.1 Preparation

##### 2.1.1.1 Construct definition

We conducted a scoping review to reveal how the Social Determinants of Mental Health are conceptualised in the literature and to ensure that no assessment tool already existed for this construct (19). This systematic literature analysis was pivotal as a well-articulated theoretical understanding is the foundation of any valid measurement (20). Based on our scoping review, we defined the Social Determinants of Mental Health as the adverse conditions in which people are born, live, work, and age, which are in turn shaped by a wider set of forces: economics, social, environmental policies, and politics. The pathway mechanisms through which the SDMentH lead to poor mental health still require further investigation. However, it is widely agreed that the effect of social determinants on mental health outcomes is mediated by the individuals’ perception of the conditions they live in and the events they are exposed to.

##### 2.1.1.2 Purposes of the assessment tool and context of use

The scoping review that we conducted did not only analyse the literature on the conceptualisation of SDMentH but also literature that dealt with the assessment of SDH (19). From this assessment focused literature, we extracted a threefold purpose for our tool: First, to increase and standardise mental health service providers’ awareness of the social and economic factors that contribute to their service users’ mental health problems. Second, to inform treatment planning. Third, to provide a means for gathering reliable epidemiological data on the SDMentH to inform policy making and research.

Research on assessing SDH in a physical health care setting demonstrated that the most effective and user-preferred assessment procedure is to complete the questionnaire electronically and discuss the responses subsequently with a health care provider (22). However, due to the lack of literature on assessing SDMentH it was impossible to determine *a priori* the exact context of use. In turn, one of the study objects was to explore the questionnaire’s exact context of use.

#### 2.1.2 Item generation

The generation of items was based on three data sources aiming to cover the SDMentH as exhaustively as possible. At this stage we followed Artino’s advice to generate more items than we were hoping to include in the final questionnaire (20).

##### 2.1.2.1 Social Determinants of Health assessment tools

The SDH and the SDMentH are conceptualised in the literature as consisting of a very similar set of social factors (19). We, therefore, included all items into our initial item pool from the SDH screening tools that were analysed in our exhaustive scoping review and/or listed in Social Needs Screening Tool Comparison Table of the Social interventions research & Evaluation Network (https://sirenetwork.ucsf.edu/tools-resources/resources/screening-tools-comparison). In total, we included items from 16 different assessment tools, see **Appendix 1** for an overview.

##### 2.1.2.2 Special section of ICD codes

A section of the ICD-10, primarily Z55-Z65, provides codes to classify “potential health hazards related to socioeconomic and psychosocial circumstances” (23). The use of those codes in clinical practice has been recommended as a way of recording SDMentH (3, 24). We chose to include this list of 246 codes for our item generation to increase the interoperability of our questionnaire by linking it to an existing international classification nomenclature (25). Thereby, we intended to facilitate the secondary usage of the date collected with the questionnaire, to inform health care planning and policy making. Only the ICD was able to serve this function, not terminologies such as SNOMED (26). In the process of our questionnaire development, we discovered that the selection of social factors for the ICD had some limitations. The methodology and rationale for inclusion of factors were not always transparent or evidence based, resulting in a limited list of social factors that affect physical and mental health (7). We addressed these limitations by widening our search bases for item generation.

##### 2.1.2.3 Literature review

Our scoping review encompassed a list of all the factors that were considered in the literature as SDMentH. Based on this list we completed our initial item pool by adding items whenever any factor had not been covered before.

In total, the initial item pool consisted of 249 items. Some of those items encompassed several SDMentHs in the form of lists [e.g.: Thinking of your childhood, have you been exposed to any of the following? Inadequate parental supervision and control (derived from ICD-10 Z62.0); Parental overprotection (Z62.1); Emotional neglect of child (Z62.4); Institutional upbringing (Z62.2)].

##### 2.1.2.4 First revision of the item pool

The research team, including two clinical psychologists (ST and PK) revised the initial item pool. We removed duplicate items that featured in several assessment tools and framed the remaining items in line with our construct definition, asking particularly for individuals’ perception of their living conditions. Furthermore, we worded all items to fit one of four item types, to make the questionnaire more easily intelligible and thereby facilitate completion: (i) binary questions assessing factors of outstanding importance such as experiences of rape or refugee status; (ii) single choice lists assessing statuses, such as employment, or education level; (iii) multiple choice lists assessing risk factors such as adverse childhood experiences; (iv) Likert scales assessing how something is being perceived: (v) Likert scales assessing frequencies, such as the number of moves within the last year. Finally, we structured the whole questionnaire, from general to more specific questions and from an economic to a social focus.

#### 2.1.3 Item selection, revision, and validation

##### 2.1.3.1 First content validation

###### 2.1.3.1.1 Recruitment

The first version of the questionnaire was validated by four mental health care service users and four mental health care professionals, none of which were part of the research team (one psychiatrist, one clinical psychologist, one social worker, one researcher in the context of SDMentH), online on the survey platform Qualtrics. The mental health care providers were selected to reflect a biopsychosocial approach to mental health, with the psychiatrist representing a biological perspective, the clinical psychologist epitomising a psychological perspective, and the social worker embodying the social standpoint. Care professionals were recruited via email, using publicly available data. Mental health care users were recruited on a self-identification basis through three Patient and Public Involvement (PPI) groups and a snowballing technique of participants inviting further potential participants. Mental health care professionals and users both have expertise in the subject matter, and both would be involved in any application of the questionnaire regarding completion and analysis. We included eight experts in accordance with guidelines for assessing content validity (27).

###### 2.1.3.1.2 Measures

Participants were asked to rate “yes” or “no” for every single item with respect to its clarity (“The item is clearly worded”), relevance (“The assessed factor is relevant for mental health”), and utility (“The information is useful for clinical practice”). At the end of the survey, they were asked for the completeness of the questionnaire. Participants had the opportunity to note their comments at any point in the survey.

We assessed the relevance of items to ensure the content validity, an essential component of any valid questionnaire (27). Moreover, we assessed the utility for clinical practice as we aimed to generate a widely applicable questionnaire (28).

###### 2.1.3.1.3 Analysis

We reworded every item that was rated as clear by fewer than seven of the eight participants, following recommendations suggested by participants wherever possible. The relevance of single items is commonly analysed in the item level Content Validity Index (I CVI), the proportion of experts rating an item as relevant (27). However, we decided to merge the relevance and utility ratings as we wanted to make sure that single items were relevant to the theoretical construct as well as useful for clinical practice. Therefore, we collapsed relevance and utility ratings into a single score out of 16. Items that received less than 13 (0.81) ratings as useful and/or relevant were removed, in line with recommendations that the I CVI should be around 0.83 (calculated by dividing the number of affirmative ratings through the total number of respective ratings) (27). When consensus was reached by the whole research team that a specific item that fell under the threshold of 13 ratings was nevertheless relevant, we revised the item. All comments provided by participants were considered for revision of the questionnaire.

### 2.2 Focus groups

#### 2.2.1 Recruitment

We recruited mental health care service users and professionals because of their expertise in the relevant field and because they are members of the target population and thereby essential to ensure content validity (29). We recruited eight mental health care users, and seven mental health care professionals, again none of which were part of the research team (one mental health nurse, one psychiatrist, two occupational therapists, two clinical psychologists, one researcher). The mental health care providers were again chosen to represent the biopsychosocial approach to mental health (mental health nurse and psychiatrist as biological, clinical psychologists as psychological, and occupational therapists as social representatives). Recruitment occurred through the same means as for the first content validation. Participants provided written consent forms prior to the focus groups.

#### 2.2.2 Procedure

Participants were sent the questionnaire, as revised following the first content validation, at least 48 hours in advance of the focus groups and were asked to read it. Participants also received a guide on how to use Zoom along with the questionnaire, as the focus groups were held on Zoom. It was left to the participants whether to turn on their cameras or to take part on an audio-only basis. Discussions were facilitated by FH and co-facilitated by IN. Focus groups were scheduled to last approximately 90 minutes, with as many breaks as desired by the participants.

#### 2.2.3 Measures

Generally, focus groups are considered a useful method for questionnaire development (20, 21). Our focus groups were semi structured, following a predefined schedule but also allowing for the discussion to develop organically.

The agenda of the single focus groups were thematically funnel shaped (30), starting with a general discussion of assessing SDMentH in clinical practice and culminating in a specific consideration of particular aspects of the questionnaire. Initially, the primary investigator welcomed everyone, defined the purpose of the focus group as gathering opinions on SDMentH and evaluating the questionnaire, and set the ground rules (i.e., everyone was always free to leave, no answers were compulsory but every contribution valuable). Subsequently, participants were asked to introduce themselves and name the most important Social Determinants of Mental Health from their perspective, encouraging everyone to speak. Afterwards, we asked about existing practices in the assessment of SDMentH. We then asked for general feedback on the questionnaire, including feedback on specific items, with respect to its relevance, clarity, utility for clinical practice, completeness, and acceptability. Furthermore, we discussed the context in which the questionnaire might be administered. During the discussions, the primary investigator probed to explore emerging perspectives in more detail, asked for suggested improvements, and tried to interconnect single contributions between the participants.

#### 2.3.4 Analysis

The focus groups were recorded, and the primary investigator generated a verbatim transcript of the records where participants were pseudonymised. Recordings were deleted after verification of the verbatim transcripts.

Analysis was guided by Fereday and Muir-Cochrane’s framework for rigorous thematic analysis (31) and conducted using the qualitative data analysis software NVivo 12. Initially we developed a coding book with predefined, deductive codes, which were meant to mark content on relevance, clarity, utility, completeness, and acceptability. The primary investigator used the initial code book on a section of the data and defined new codes to capture data that was not covered by the initial codes. These deductive and inductive codes were then discussed with the whole research team and applied to parts of the data to ensure reliability. The agreed codebook was then applied to the whole data set. Subsequently, we connected the codes to identify themes. Finally, we made sure that the resulting themes were strictly linked to the original data, by scrutinising all foregoing steps.

We used the themes to inform the format of the entire questionnaire, the wording of single items, and the context of administration. Based on this analysis, the tool was revised by the primary investigator and discussed by the research team.

### 2.3 Second content validation

The second content validation was conducted in the same way as the first content validation. Participants were selected based on identical inclusion criteria through the same means of recruitment. No participants that took part in previous steps of the questionnaire development were eligible. The same measures as in the first content validation were again applied on Qualtrics. Analysis was exactly as in the first round.

## 3 Results

### 3.1 First content validation

Recruitment occurred between the 28^th^ of July 2021 and the 12^th^ of November 2021.

16 items fell under the threshold pertaining to the relevance and utility ratings, 14 of which were removed and two reworded. 70 items received fewer than seven ratings as clear and were reworded in consequence. One item was added, following the suggestion of one participant.

Several ratings were missing, presumably due to participants deciding to only indicate their disagreement instead of demonstrating their approval for each item on three measures (relevance, utility, clarity). However, following the predefined revision procedure, we revised items whenever the predetermined approval threshold was in doubt.

The comments revealed several repeating issues. One common criticism pertained to the quantifying answer options. Participants criticised that the answer options ranging from *frequently* to *never* would not be precise enough. However, in line with the construct definition, the questionnaire was not intended to assess the actual number of moves for examples, but whether an individual perceived them as too frequent. Following the criticism, we explicated this more clearly in the introduction of the questionnaire. Moreover, participants called for more contextualisation as to why certain factors would be assessed. In response to this, we reworded items, for example, along the lines of “One social factor that can have an impact on mental health is whether one is part of the ethnic majority or part of the ethnic minority. Do you belong to the same ethnicity as the majority around you?”. Furthermore, participants suggested, for several items, adding specific problems within the lists of potential problems. For example, participants suggested to include “Physical appearance” as one potential factor about which people might experience discrimination. Generally, participants recommended simplification of many items. Participants also suggested increasing the clarity of items by defining the object of interest better or adding more examples. In this vein we, for example, defined wealth in one item as “comprising of savings, stocks, assets, property, pension, etc”. In a different item we introduced an exemplification of formally organised groups “(like a political party or a sports team)”.

After revisions based on the first content validation, the questionnaire consisted of 85 items.

### 3.2 Focus groups

Recruitment for the focus groups started on the 6^th^ of January 2022. Eight mental health care service users were willing to participate in one focus group, but only seven attended the zoom meeting on the 1^st^ of February. It was not possible to find one date to meet with all mental health care providers that had agreed to participate. Hence, we conducted one focus group with one clinical psychologist, one researcher, and two occupational therapists on the 23rd of March 2022 and another focus group with one psychiatrist, one clinical psychologist, and one mental health nurse on the 5^th^ of April 2022. All focus groups lasted approximately 90 minutes and all participants stayed for the entire meetings. All but two participants took part with video. None of the participants showed any signs of distress. The focus groups revealed seven themes that are summarised in **Table 2**.

**Table 2:** Themes in the focus groups and consequences to the questionnaire.

Theme	Contribution Focus group 1	Contribution Focus group 2	Contribution Focus group 3	Inferences pertaining the questionnaire
Current practices of assessing social contextual information	Social contextual information is currently not sufficiently assessed. Therapy focuses on thoughts about living conditions instead of on the living conditions themselves.	Such information is only assessed if the service user presents certain risk factors. Single professions only focus on those aspects that fit their professional remit. No standardised protocol.	One participant says there would be streamlined and exhaustive assessments in place. Other two say that the social history of service users used to be assessed in more detail and is now mostly being ignored. Any assessment of social contextual information is risk and referral focused and the write up time-consuming.	The absence of a (standardised) assessment in current clinical practice further justifies the need for an assessment tool. The time-consuming write up is an argument in favour of a multiple-choice format.
Control over data	Participants express concerns about the potential for the data to be misinterpreted without enough contextualisation. Participants want to have the opportunity to explain their responses and want to choose which data to share.			Each item is now accompanied by a tick box to indicate whether respondents deem the assessed factors to be particularly relevant to their mental health. This is meant to empower respondents with respect to the interpretation of their responses. We also included an open text box at the end of every domain for participants to explain their given responses.
Excluding and stereotyping rigid format of multiple-choice	The multiple-choice format does not cover individuality and may appear as a checklist to prove that one deserves help. The categories have the potential to stereotype and other respondents.	A conversational style, including open questions, elucidates more information and is less in risk of sounding accusatory.		The included open text boxes at the end of each domain provide the chance to capture individuality. We also revised the wording of all questions to be more inclusive, to prevent people from feeling othered or forced into stereotypes.
Benefits of assessing SDMentH	Helps to understand the service user better. Informs referrals and prevention planning.	Enables holistic care, and treatment of the causes rather than symptoms. Can provide justification for care and improve the use of health care resources.	Identifying underlying causes, tailoring treatment and referrals. Improves therapeutic alliance.	Provides further justification for the questionnaire.
Risks of assessing SDMentH	Might make service users more vulnerable. Impossible to address the SDMentH in an already overstretched health service.	Raising hopes that cannot be met (opening Pandora’s box). Risking the doctor-patient relationship as the questions might be perceived as out of scope/accusatory.	Not feasible in time pressured services and risk of raising expectations that cannot be met.	Tried to set the expectations very clear in the introduction of the questionnaire that service providers will not be able to help with all of the assessed factors. Also, shortened the questionnaire to make it more compatible with given time limitations in clinical practice.
Characteristics of ideal assessment of SDMentH	The person administering the questionnaire should be a trusted medical professional. Qualitative descriptions of individual experiences should be captured by the questionnaire.	Ideally the assessment would be adapted to the needs of the individual respondent, allowing for a conversational style, and focus on changeable determinants. Primary care would be a privileged setting for administering.	The assessment should be at the beginning of someone’s mental health care journey. It should follow the person through different services without getting lost in transitions. Secondary care would be best suited for administering the questionnaire.	Due to contradicting recommendations about the best suited setting for administering the questionnaire, we left it open to any service using the questionnaire to decide on the concrete setting.

### 3.3 Thematic analysis

#### 3.3.1 Focus group 1

Focus group 1, with mental health care users, primarily centred around three main themes: *the risks and benefits of assessing social contextual information in clinical practice*; *control over data; excluding and stereotyping rigid format*.

Potential risks of an SDMentH were identified; the main one being that the English mental health care services would not have capacity to take on new responsibilities (Participant 1 “How on earth can an underfunded, under-resourced mental health service affect people’s housing situation?”). Current practice was said to ignore the conditions people are living in and instead focus on changing people’s thoughts about their lives. Potential benefits of an improved assessment were that it could inform referrals and prevention and that mental health care providers would get a better understanding of the service users (Participant 3 “to identify, what set them on the journey that led them to where we are today and what can we learn from that”).

*The control over data* theme had several aspects. On the one hand, participants expressed concerns about the risk of inappropriate interpretation and use of completed questionnaires (Participant 1 “This information could be used in ways that make people more vulnerable”; Participant 2 “It is not just objective data and people come to it with their prejudices and their understanding of how the world works”). Closely linked to this was the issue of who would administer the questionnaire (Participant 4 “So, simply put it depends on who is asking me that questionnaire. And there is that element of trust”). Participants stated that one way of getting more control over their data would be to provide the chance to explain their responses (Participant 2 “And then actually giving people just that little box and let them just explain why they have given the answer that they have”).

The *excluding and stereotyping rigid format* theme was closely associated with the *control over data* theme. The multiple-choice format of the questionnaire was criticised as excluding people, since the questionnaire appeared to list criteria that had to be fulfilled to prove worthy of help (Participant 5 “The reason why you experience these issues should be one of the issues, so if you don’t find yourself represented there, then perhaps, you don’t belong in this process”). Furthermore, the format was criticised for disabling qualitative descriptions. Moreover, participants pointed out that the format of the questionnaire might stereotype people (Participant 4 “They pigeonhole me into boxes, or people into boxes, so you fit into these boxes”).

#### 3.3.2 Focus group 2

Analysis of focus group 2 revealed three main themes; *risks and benefits of assessing SDMentH; characteristics of an ideal assessment; professional remits in addressing and assessing SDMentH.*

Proposed risks of an SDMentH assessment were that it could open expectations that cannot be met in mental health care services; to “open Pandora’s box”, as some participants called it. Another discussed risk was that posing the questions on the questionnaire could sound accusatory and by that endanger the relationship between mental health care service providers and users (Participant 8 said that users might think “You are trying to take over my life, you are concerning yourself with something that does not concern you”). Considered benefits were that treatment could become more holistic through an SDMentH assessment and start to address the causes of poor health rather than the symptoms. Additionally, such an assessment could justify further treatment in some cases (Participant 9 gives the example of team meetings pertaining to discharge “actually they can’t go home, because of, you know familiar problems”).

One of the characteristics of an ideal assessment was a conversational style including open questions (Participant 10 “Different styles work for different professions best, but the less open your questions are, the less information you sometimes get”). Questions should focus on changeable determinants and ideally there should be services available to refer to, in response to given answers. Furthermore, enough time to complete and discuss the questionnaire was said to be essential, as well as a trusting relationship between the person completing the questionnaire and the person administering and interpreting it. Moreover, the person administering it should be well trained in assessing sensitive information. The environment in which the questionnaire is completed should be supportive and controlled. Primary care was stated to be the privileged setting for administering the questionnaire, as this setting encourages longer lasting relationships between health care service providers and users.

The professional remits were repeatedly mentioned as an issue in the assessment of social contextual information. A problem would arise as the SDMentH span multiple disciplines, but single professions would only focus on very specific areas (Participant 11 “So the nurse would be asking about psychological health and medication and the social worker would focus very much on family and housing and so on”).

#### 3.3.3 Focus group 3

The discussion in focus group 3 was mainly around *current practices in assessing social contextual information*; *benefits of assessing SDMentH; characteristics of an ideal assessment*.

One participant (mental health nurse) said that the SDMentH would be assessed in a thorough, streamlined way in emergency departments. The write up of those assessments would often take up more time than the assessment itself. Other participants stated that such an assessment used to form part of their routine but that it would be insufficiently done in current practice (Participant 15 “In term of biopsychosocial formulation, the social bit is the bit that is really missed out in psychiatry”). Conducted assessment appeared to be risk and referral focused (Participant 15 “And it tends to be a quick-fire measurement of risks”).

Raised benefits of an SDMentH assessment were that it would identify underlying causal explanations, and tailor treatment for individuals (Participant 14 “Well, I think a better social assessment would allow us to target a person much better”). One proposed side-effect of this improved understanding of a service user is that the alliance between provider and user would strengthen.

Time was considered to be an important aspect for the ideal assessment. First, the assessment would have to stand at the beginning of someone’s mental health journey, as it would otherwise not be assessed to a later point. Second, multiple services lack the available time to administer the questionnaire, such as already overloaded GPs, or crisis teams. A recommended setting was psychiatric inpatient units, given that this setting facilitates availability and staff would have the training to follow up on responses. Another critical aspect discussed was that information on service users often gets lost in the transition between different services, such as youth and adult services. The ideal SDMentH assessment would follow the service user through different services so that service users would not have to repeatedly provide the same information.

#### 3.3.4 Overview of all focus groups

Most participants stated that SDMentH are not currently sufficiently assessed in clinical practice. Participants agreed that such an assessment would improve the understanding of individual service users and would have the potential to reveal underlying causes of poor mental health. This would inform better targeted and more holistic treatment. One participant argued that the relationship between providers and users would benefit from such an assessment, however other participants posited that this relationship might suffer as some questions could be perceived as accusatory. Other risks identified were that already overloaded mental health care services would be unable to address the social contextual factors of service users. It has been criticised that assessing SDMentH would open Pandora’s box of adverse factors that cannot be addressed in clinical practice.

With respect to the ideal characteristics of an SDMentH assessment, there was a consensus across the focus groups that a trusting relationship between the person completing the questionnaire and the person interpreting the responses would be fundamental. In addition, there was agreement that the questions should focus on changeable factors. Furthermore, control over the data of the assessed person was raised as an important issue, as well as the need for space providing the opportunity to qualitatively describe idiosyncratic experiences besides in a rigid multiple-choice format. Participants disagreed on the most suitable setting for the assessment, some arguing in favour of primary care settings due to the long persisting relationship between providers and users, while others favoured secondary care, due to a greater expertise of staff and more concentrated time.

#### 3.3.5 Inferences for the questionnaire

Participants in the focus groups commented on specific items. All these comments have been considered for the revision of the questionnaire. For example, several participants criticised the initial item as too broad and we removed it consequentially (“In your experience, are you treated as a free human being? Very free/Rather free/Rather unfree/Very unfree”).

On a more principal level, the thematic analyses led to five changes to the questionnaire. First, due to the emphasised importance of time in a stretched health service we shortened the questionnaire as much as we could without losing any of the assessed factors. We focused on removing redundancy and simplifying wording.

Second, in response to the raised concern that a SDMentH assessment could facilitate stereotyping and making people more vulnerable, we revised the whole questionnaire to make the language more inclusive. For example, we reworded “… whether one is part of the ethnic majority” to “… whether we identify with being part of the ethnic majority”.

Third, given the disagreement pertaining to the best suited setting for the assessment we left aspects of the administration open to potential services administering the questionnaire. The introduction therefore has one uncompleted section: “What happens with the completed questionnaires? (This section will be completed by the single health care settings applying this questionnaire.)”.

Fourth, the focus groups revealed the importance of respondents feeling in control over their responses and how they are interpreted. At questionnaire level we therefore included a further statement with a tick box to accompany each item, reading “*This is particularly important to my mental health, and I would like to discuss this further”*. One participant in the focus group 1 suggested something along this line. The intention was to enable respondents to prioritise the assessed factors and to interpret their own answers.

Fifth, the focus groups demonstrated the dangers of a rigid multiple-choice format that excludes qualitative descriptions. We, therefore, included free writing spaces at the end of every section, for example, “Is there anything you want to say about the answers you have given to the questions in this block? Or, is there anything you would like to add about how your (potential) minority status and experiences of discrimination affect your mental health?”. This was intended to enable respondents to explain what has not been covered by the multiple-choice format and to provide respondents with more control over their responses.

**Table 2** provides an overview of the themes revealed by the focus groups and the inferences for the questionnaire.

The revised questionnaire consisted of 76 items.

### 3.4 Second content validation

Recruitment occurred between the 23^rd^ of November 2022 and the 28^th^ of February 2023.

None of the items fell under the threshold for relevance and utility. 22 items received fewer than seven ratings as clear. Based on the comments we revised 49 items, removed two items and merged two items with other existing items. Overall, the scale level content validity index based on the average method was 0.95, representing a good content validity (27).

We removed two items even though they were above the threshold for utility and relevance ratings because the comments indicated that the content of those items would be covered by other items. Moreover, the general feedback of a few participants was that the questionnaire would need to be shortened.

There were several issues that were repeatedly discussed in the comments. First, the item types that were introduced following the focus groups (“This is particularly important to my mental health, and I would like to discuss this further” and *“*Is there anything you want to say about the answers you have given to the questions in this block? Or, is there anything you would like to add about how your (potential) minority status and experiences of discrimination affect your mental health?”) were consistently praised by participants. Second, the answer options containing the word “rather” were criticised as not very accessible. In the revision of these answer options, we followed the advice of participants and replaced “rather” with “somewhat” in the revision of 29 items. Third, a number of items that assessed a status were criticised as not encompassing an exhaustive list of options. For example, the housing status item did not enable respondents to declare that they would not have private housing but were living in institutions such as prison or hospital. In all these instances we followed the suggestions of participants. Fourth, it was suggested that items that contained an “other”- answer option should provide the opportunity to specify this other option in free writing columns. We implemented this recommendation. See **Appendix 2** for the final version of the questionnaire.

## 4 Discussion

### 4.1 Summary of findings

This study developed and content validated a questionnaire for assessing the Social Determinants of Mental Health (SDMentH) in clinical practice. Several tools for assessing the Social Determinants of Health (SDH) already exist (18), but it would be theoretically inappropriate to use those tools in a mental health context given the aetiological and epistemological differences between the constructs of Social Determinants of (physical) Health (SDH) and Social Determinants of Mental Health that we found in a scoping review (19). We aimed to develop a questionnaire for assessing the SDMentH to facilitate a more holistic mental health care.

The development of the questionnaire occurred in three stages: (i) the preparation including a clear definition of the SDMentH, (ii) initial item generation, which was based on a.) existing tools to assess the SDH, b.) a list of all SDMentH considered in the literature as revealed by a scoping review (19), and c.) the ICD codes to record social contextual information, (iii) item selection, validation, and revision, based on surveys and focus groups with mental health care service users and professionals. When the questionnaire entered the third stage it included 249 items, which were reduced to 73 with the help of 15 mental health care professionals and 15 mental health care service users in total.

The questionnaire covers 8 domains: (i) Minority status and discrimination; (ii) Education and employment; (iii) Income, wealth, financial strain; (iv) Access to healthcare and food; (v) Neighbourhood, transportation, housing; (vi) Social network, friends, family, and caring responsibility; (vii) Adverse experiences in childhood and adulthood; (viii) Losses, life events, and physical health.

All participants confirmed that a questionnaire for the SDMentH would be principally useful and relevant in mental health care and that currently no comparable questionnaire exists. There was consensus that such a questionnaire would ideally inform treatment planning, increase understanding of mental health care service users, and enable preventive interventions. The most commonly cited risk of implementing a SDMentH questionnaire was that it might raise expectations that cannot be met in mental health care services. Our study revealed contradicting views on the ideal administration context for the questionnaire. Consequently, we did not specify this context but left it for respective services to decide on how they wanted to administer the questionnaire. Moreover, participants disagreed on the effect of administering the questionnaire on the relationship between service providers and users.

### 4.2 Strengths and limitations

The main strength of this questionnaire is that its systematic development complied with relevant guidelines (20, 21). This questionnaire has a good theoretically grounding from a preceding systematic literature review and is content validated by members of the target population, i.e., mental health care service users and professionals This distinguishes this questionnaire from most existing assessment tools for Social Determinants of Health, which are regularly found to be built on an insufficient theoretical foundation and to lack validation by practitioners and patients (8, 32, 33).

Another methodological strength of this study is the thorough content validation of the questionnaire, in two rounds of an online survey and three focus groups. This goes beyond basic requirements for content validation (27) and ensures a more robust validation. Furthermore, in our content validity index we not only considered whether single items were rated as relevant for the construct but also whether items were rated as useful for clinical practice. Therefore, the single items and the whole questionnaire bear relevance for the construct of SDMentH and for the context of application.

A strength of our recruitment was that it depicted the multifaceted perspectives and actors of mental health care. At every stage of validation, we included representatives of all three components of the biopsychosocial model, i.e., psychiatrists and mental health nurses for the biological approach, clinical psychologists for the psychological aspect, and occupational therapists and social workers for the social component of the model. Including as many service users as service professionals guaranteed that the voices of the most affected were heard, applying equality principles that are intrinsic to any SDMentH research at a study design level.

One limitation of this study is that we did not link the assessed SDMentH to support resources. Such a link is required (34) to ensure that positive risk results are followed up with referrals to adequate help. This *screening and referral paradigm* however has recently been criticised for being reductionistic and not taking all aspects of treatment into account (35). Collecting SDMentH data can be beneficial even if not all assessed factors can be readily remedied by a specialist service. Our focus groups revealed that the assessment of SDMentH can strengthen the provider-user relationship, support providers’ understanding of users, and in the long term improve aetiological models and increase social investment, most of which supported by Byhoff and Gottlieb (35). To be transparent about limitations, we acknowledge in the introduction of the questionnaire that the mental health care service can only be of limited direct help for many of the assessed factors. A further limitation is that our recruitment was open to a selection bias. Most of the mental health care providers that participated in the evaluation of the questionnaire were known to the research team and had an interest in psychosocial perspectives of mental health problems. It is possible that more biomedically oriented professionals would be less validating of the purpose of the questionnaire.

### 4.3 Future research

The psychometric properties of this questionnaire must be tested in further studies. To this purpose the questionnaire should be administered to a pilot sample of mental health care service users. Based on the completed questionnaires it will be possible to establish the reliability. Particularly important here is the test-retest reliability to ensure that the questionnaire can be used in longitudinal studies. Moreover, an exploratory factors analysis could identify underlying factors and thereby pinpoint ways to further condense the questionnaire. In line with recent guidelines, we recommend a sample size greater than 400 to enable valid inferences from the exploratory factor analysis (36). Recruiting a diverse sample with respect to the assessed Social Determinants of Mental Health will be critical to capture the full range of potential responses. Based on the pilot sample the questionnaire could also be standardised, to distinguish this questionnaire from most SDH screening tools that are not standardised (18). Furthermore, a feasibility study must test whether this questionnaire is implementable into busy clinical practice.

Our focus groups revealed contradicting recommendations as to the most suitable context in which to assess the SDMentH. The characteristics of an ideal assessment of social contextual information in mental health care settings is currently understudied, especially in the UK. More research is needed to determine the most helpful point in someone’s mental health care journey to assess the SDMentH.

Our focus groups also suggested contradictory views on the potential effects of the SDMentH questionnaire on the relationship between service providers and users. Against the backdrop of these conflicting theoretical arguments, the effects should be empirically tested.

### 4.4 Implications

This study evidenced the need for a standardised tool to assess mental health care service users’ social contextual information and generated a questionnaire to this purpose. The questionnaire rests on a well-articulated theoretical basis and has been validated by members of the target population, i.e., mental health care professionals and users. Qualitative and quantitative evaluation of the questionnaire indicated that implementing the questionnaire into clinical practice would contribute to a more holistic mental health care service provision that tackles the underlying reasons for poor mental health. Future studies must test the reliability of the questionnaire and whether it is feasible to implement it into clinical practice. Furthermore, more research is needed into the optimal context of assessing the SDMentH.

## Data availability statement

The raw data supporting the conclusions of this article will be made available by the authors, without undue reservation.

## Ethics statement

The studies involving humans were approved by The University of Manchester UREC. The studies were conducted in accordance with the local legislation and institutional requirements. The participants provided their written informed consent to participate in this study.

## Author contributions

FH: Conceptualization, Data curation, Investigation, Methodology, Writing – original draft. PK: Formal analysis, Supervision, Writing – review & editing. IN: Investigation, Methodology, Writing – review & editing. ST: Formal Analysis, Supervision, Writing – review & editing.
