# Supplementary material for: Development and content validation of a questionnaire to assess the social determinants of mental health in clinical practice
Source: Front Psychiatry. 2024 May 20;15:1377751. doi: 10.3389/fpsyt.2024.1377751 (PMC11145063; doi:10.3389/fpsyt.2024.1377751)
Supplement: Supplementary file 1 [file Table_1.docx]

## Appendix 1: Screening tools used for item generation

| **Name of the tool** | **Source and availability of the tool** |
| --- | --- |
| AccessHealth Spartanburg: Social Determinants Screening Tool | Available under: <https://www.chcs.org/media/AccessHealth-Social-Determinant-Screening_102517.pdf>  Discussed in: Thomas-Henkel, C., & Schulman, M. (2017). Screening for social determinants of health in populations with complex needs: implementation considerations. *Center for Health Care Strategies*, *10*. |
| Accountable Health Communities Core Health-Related Social Needs Screening Questions | Billioux, A., Verlander, K., Anthony, S., & Alley, D. (2017). Standardized screening for health-related social needs in clinical settings: the accountable health communities screening tool. *NAM perspectives*. |
| Arlington Screening Tool- Pilot Questions | 2020, available under: https://sirenetwork.ucsf.edu/sites/default/files/2021-02/Arlington%20Screening%20Tool-%20Final%20version.pdf |
| Family wellness screen | Uwemedimo, O. T., & May, H. (2018). Disparities in utilization of social determinants of health referrals among children in immigrant families. *Frontiers in pediatrics*, *6*, 207 |
| Health Begins' Upstream Risks Screening Tool | Hensley, C., Joseph, A., Shah, S., & O’Dea, C. (2017). Addressing social determinants of health at a federally qualified health center. *International Public Health Journal*, *9*(2), 189. |
| IOM Social and Behavioural domains and measurement | Committee on the Recommended Social and Behavioral Domains and Measures for Electronic Health Record. (2014). *Capturing social and behavioral domains in electronic health records: phase 1*. National Academies Press. |
| iScreen Social Screening Questionnaire | Gottlieb, L., Hessler, D., Long, D., Amaya, A., & Adler, N. (2014). A randomized trial on screening for social determinants of health: the iScreen study. *Pediatrics*, *134*(6), e1611-e1618. |
| Kaiser Permanente's Your Current Life Situation (YCLS) | 2017, available under:  https://sirenetwork.ucsf.edu/sites/default/files/Your%20Current%20Life%20Situation%20Questionnaire%20v2-0%20%28Core%20and%20supplemental%29%20no%20highlights.pdf |
| Medical Legal Partnership | 2014, available under: https://medical-legalpartnership.org/screening-tool/ |
| North Carolina Medicaid Screening Tool | North Carolina Department of Health and Human Services. *Using Standardized Social Determinants of Health Screening Questions to Identify and Assist Patients with Unmet Health-related Resource Needs in Health and Human Services [Internet].* Raleigh, NC: North Carolina Department of Health and Human Services; (2018) |
| Pilot Social Health History Screening Tool Research Project Questionnaire | Browne-Yung, K., Freeman, T., Battersby, M., McEvoy, D. R., & Baum, F. (2019). Developing a screening tool to recognise social determinants of health in Australian clinical settings. *Public Health Research & Practice*, *29*(4), e28341813 |
| Protocol for Responding to & Assessing Patients’ Assets, Risks & Experiences (PRAPARE) | National Association of Community Health Centers. (2016). PRAPARE implementation and action toolkit. |
| Social Determinants of Health Screening Tool  Thrive | de la Vega, P. B., Losi, S., Martinez, L. S., Bovell-Ammon, A., Garg, A., James, T., ... & Kressin, N. R. (2019). Implementing an EHR-based screening and referral system to address social determinants of health in primary care. *Medical care*, *57*, S133-S139 |
| Structural vulnerability Assessment tool | Bourgois, P., Holmes, S. M., Sue, K., & Quesada, J. (2017). Structural vulnerability: operationalizing the concept to address health disparities in clinical care. *Academic medicine: journal of the Association of American Medical Colleges*, *92*(3), 299. |
| Total Health Assessment Questionnaire for Medicare Members | 2017, available under https://sirenetwork.ucsf.edu/sites/default/files/Medicare%20THA%20questionnaire%20v2%20%28rvd%2012-5-14%29%20with%20Sources.pdf |
| WellRX tool kit, | Page-Reeves, J., Kaufman, W., Bleecker, M., Norris, J., McCalmont, K., Ianakieva, V., ... & Kaufman, A. (2016). Addressing social determinants of health in a clinic setting: the WellRx pilot in Albuquerque, New Mexico. *The Journal of the American Board of Family Medicine*, *29*(3), 414-418. |
