# Supplementary material for: Development and content validation of a questionnaire to assess the social determinants of mental health in clinical practice
Source: Front Psychiatry. 2024 May 20;15:1377751. doi: 10.3389/fpsyt.2024.1377751 (PMC11145063; doi:10.3389/fpsyt.2024.1377751)
Supplement: Supplementary file 2 [file Table_2.docx]

## Appendix 2: Clinical questionnaire for the social determinants of mental health

**Clinical questionnaire for the social determinants of mental health**

What is the object and purpose of this questionnaire?

It is well known that social, political, and cultural factors impact on our mental health (how we think and how we feel). These include issues such as housing (whether we have a roof over our head or not), employment (whether we have a rewarding and secure job), but also things like government services (whether we receive the support we need), our neighbourhood, whether we feel safe and supported. Understanding the things that are relevant in our lives may help clinicians inform and improve the care we receive.

The questionnaire does not contain a complete list of all social factors that affect mental health. Instead, the questionnaire asks about events and conditions that are most commonly reported as being relevant for mental health. Our mental health, however, is very personal and depends on a variety of individual factors. Therefore, some social factors that are most influential to your mental health might not be explicitly mentioned. There will be opportunities to write down those factors that are relevant to your mental health but are not listed in the questionnaire.

A trigger warning and how to complete the questions.

Some of the questions touch on very sensitive topics such as abuse experiences and your financial situation. You do not have to answer every question, but any answer will be informative and will contribute to improving the care you will receive.

We are interested in your individual perspective. There are no right or wrong answers. What is important is that you answer in the most honest way you can. For example, some questions ask you how often certain experiences occur with a choice of “frequently”; “sometimes”; “rarely”; “never”. For these questions we are interested in how things generally appear to you, rather than any specific numbers.

All questions are informed by research and clinical experience in the field of mental health care.

You will have the opportunity to discuss all the questions of this questionnaire. However, if you want to talk about something in particular, please indicate this by ticking the box below the question.

What happens with the completed questionnaires?

(This section will be completed by the single health care settings applying this questionnaire.)

**Minority status and discrimination**

1. One social factor that can have an impact on our mental health is whether we identify as being part of the ethnic majority or part of an ethnic minority. Do you belong to the same ethnicity as the majority around you?

“*I belong to the same ethnicity as…”* (Please check **all** that apply)

| 1*. most people in* *the* ***country*** *I am living in.”* | 2. *most people in the* ***neighbourhood*** *I am living in.”* | 3. *most people in my* ***work environment/ school environment****.”* | *4. most people in my* ***group of friends.”*** | 5. **v*ery few*** *people around me.”* |
| --- | --- | --- | --- | --- |

*□ This is particularly important to my mental health, and I would like to discuss this further*

1. Do you share the same culture (norms, values, beliefs) as most people around you?

*“I share cultural norms, values, beliefs, with …”* (Please check **all** that apply)

| *1. most of the people in the* ***country*** *I am living in.”* | *2. most of the people in the* ***neighbourhood*** *I am living in.”* | 3*. most of the people* *in* *my* ***work environment/ school environment****.”* | *4. most of the people in* ***my group of friends****.”* | 5. **v*ery few*** *people around me.”* |
| --- | --- | --- | --- | --- |

*□ This is particularly important to my mental health, and I would like to discuss this further*

1. Are you, or have you ever been, an immigrant or refugee? (Please check **all** that apply)

| Immigrant |  |
| --- | --- |
| Refugee |  |
| Never been an immigrant or refugee |  |

*□ This is particularly important to my mental health, and I would like to discuss this further*

If immigrant or refugee before:

1. How worried are you about your or a family member’s immigration status?

| 1. Very worried | 2. Somewhat worried | 3. Hardly worried | 4.Not worried at all | 5. Not applicable |
| --- | --- | --- | --- | --- |

*□ This is particularly important to my mental health, and I would like to discuss this further*

1. There are expectations in societies about how people of a certain gender should behave or appear. How worried are you about meeting the expectations that are associated with the gender you identify with?

| 1. Very worried | 2. Somewhat worried | 3. Hardly worried | 4. Not worried at all |
| --- | --- | --- | --- |

*□ This is particularly important to my mental health, and I would like to discuss this further*

1. Have you ever been subject to discrimination based on any of the following factors? (Please check **all** that apply)

| Race |  |
| --- | --- |
| Gender |  |
| Sexual orientation |  |
| Disability |  |
| Mental health |  |
| Age |  |
| Physical appearance |  |
| Religion |  |
| Social class |  |

| Other: |  |
| --- | --- |

*□ This is particularly important to my mental health, and I would like to discuss this further*

1. Is there anything you want to say about the answers you have given to the questions in this block? Or, is there anything you would like to add about how your (potential) minority status and experiences of discrimination affect your mental health?

**Education and employment**

1. What is the highest level of schooling you have completed? (Please choose **one** of the following options)

| No formal education |  |
| --- | --- |
| Primary school |  |
| Secondary school or equivalent |  |
| Sixth form/ upper school or equivalent |  |
| Undergraduate courses |  |
| Post graduate courses |  |

*□ This is particularly important to my mental health, and I would like to discuss this further*

1. How do you experience getting access to the education you want?

| 1. Very difficult | 2. Somewhat difficult | 3. Somewhat easy | 4. Very easy |
| --- | --- | --- | --- |

*□ This is particularly important to my mental health, and I would like to discuss this further*

1. How satisfied are you with your ability to read?

| 1. Very satisfied | 2. Somewhat satisfied | 3. Somewhat dissatisfied | 4. Very dissatisfied |
| --- | --- | --- | --- |

*□ This is particularly important to my mental health, and I would like to discuss this further*

1. How satisfied are you with your ability to speak English?

| 1. Very satisfied | 2. Somewhat satisfied | 3. Somewhat dissatisfied | 4. Very dissatisfied |
| --- | --- | --- | --- |

*□ This is particularly important to my mental health, and I would like to discuss this further*

1. Which best describes your main current occupation? (Please choose **one** of the following options)

| Engaged in full-time education (pupil/ student/ apprentice) |  |
| --- | --- |
| Homemaker, not working outside the home |  |
| Employed (or self-employed) full-time |  |
| Employed (or self-employed) part-time |  |
| Employed but temporarily away from job |  |
| Involved in regular volunteer work |  |
| Not employed/ Laid off 6 months or less |  |
| Not employed/ Laid off more than 6 months |  |
| Not/ partially employed due to a disability |  |
| Retired |  |

*□ This is particularly important to my mental health, and I would like to discuss this further*

If not employed in any form

B.-1) How worried are you about finding a job?

| 1. Very worried | 2. Somewhat worried | 3. Hardly worried | 4. Not worried at all | 5. Not applicable |
| --- | --- | --- | --- | --- |

*□ This is particularly important to my mental health, and I would like to discuss this further*

If employed in some form:

B.-2) Thinking of your work-environment, are you exposed to any of the following problems? (Please check **all** that apply)

| Radiation |  |
| --- | --- |
| Accessibility issues |  |
| Unsociable working hours (outside of 9am to 5pm) |  |
| Precarious working conditions (Zero-hours contract etc.) |  |
| Noise |  |
| Toxic air contaminants |  |
| Other toxic agents |  |
| Conflict with boss or work mates |  |

| Other |  |
| --- | --- |

*□ This is particularly important to my mental health, and I would like to discuss this further*

C.2) How satisfied are you with your present job or business in terms of job security?

| 1. Very satisfied | 2. Somewhat satisfied | 3. Somewhat dissatisfied | 4. Very dissatisfied |
| --- | --- | --- | --- |

*□ This is particularly important to my mental health, and I would like to discuss this further*

D.2) How satisfied are you with the social status that comes with your job? Social status is defined as prestige, respect, and esteem in the eyes of others.

| 1. Very satisfied | 2. Somewhat satisfied | 3. Somewhat dissatisfied | 4. Very dissatisfied |
| --- | --- | --- | --- |

*□ This is particularly important to my mental health, and I would like to discuss this furthe*

E.2) Thinking of the characteristics of your job, which of the following statements describe your situation? (Please check **all** that apply)

| *I am underpaid compared to those with my level of knowledge, skills.* |  |
| --- | --- |
| *My rank at work is less than it should be for someone with my ability.* |  |
| *The work I do is very important to me.* |  |
| *My job activities are personally meaningful to me.* |  |
| *None of the above.* |  |

*□ This is particularly important to my mental health, and I would like to discuss this further*

1. Have you ever served in the military? (Please choose **one** of the following options)

| Yes, discharged or dismissed |  |
| --- | --- |
| Yes, currently serving in the military |  |
| No |  |

*□ This is particularly important to my mental health, and I would like to discuss this further*

1. Is there anything you want to say about the answers you have given to the questions in this block? Or, is there anything you would like to add about how your education and employment affect your mental health?

**Income, wealth, financial strain**

1. How satisfied are you with your income - the total sum of annual earnings - compared to other people in the UK?

| 1. Very satisfied | 2. Somewhat satisfied | 3. Somewhat dissatisfied | 4. Very dissatisfied |
| --- | --- | --- | --- |

*□ This is particularly important to my mental health, and I would like to discuss this further*

1. How satisfied are you with your wealth – comprising of savings, stocks, assets, property, pension, etc. - compared to other people in the UK?

| 1. Very satisfied | 2. Somewhat satisfied | 3. Somewhat dissatisfied | 4. Very dissatisfied |
| --- | --- | --- | --- |

*□ This is particularly important to my mental health, and I would like to discuss this further*

1. Within the past 12 months, did you ever struggle to pay for any of the following basics:

| Food |  |
| --- | --- |
| Clothes |  |
| Accommodation |  |
| Medical care |  |
| Heating |  |

*□ This is particularly important to my mental health, and I would like to discuss this further*

1. If you have any debts, how worried are you about your debts?

| 1. Very worried | 2. Somewhat worried | 3. Hardly worried | 4. Not worried at all | 5. Not applicable |
| --- | --- | --- | --- | --- |

*□ This is particularly important to my mental health, and I would like to discuss this further*

1. Do you receive any form of welfare support, like Personal Independence Payments (PIP), housing benefit, universal credit, disability living allowance, etc.? (Please choose **one** of the following options)

| Yes, and the assistance I receive is sufficient | Yes, and the assistance I receive is insufficient | No |
| --- | --- | --- |

*□ This is particularly important to my mental health, and I would like to discuss this further*

1. Generally, how do you experience getting access to the welfare support you need?

| 1. Very difficult | 2. Somewhat difficult | 3. Somewhat easy | 4. Very easy |
| --- | --- | --- | --- |

*□ This is particularly important to my mental health, and I would like to discuss this further*

1. Is there anything you want to say about the answers you have given to the questions in this block? Or, is there anything you would like to add about how your income, assets, and benefits affect your mental health?

**Access to healthcare and food**

1. Generally, how do you experience getting access to the physical health care you need?

| 1. Very difficult | 2. Somewhat difficult | 3. Somewhat easy | 4. Very easy |
| --- | --- | --- | --- |

*□ This is particularly important to my mental health, and I would like to discuss this further*

1. Generally, how do you experience getting access to mental health care services (provided for example by a counsellor, mental health nurse, psychologist, or psychiatrist)?

| 1. Very difficult | 2. Somewhat difficult | 3. Somewhat easy | 4. Very easy |
| --- | --- | --- | --- |

*□ This is particularly important to my mental health, and I would like to discuss this further*

1. Generally, what is your experience of following your doctor’s treatment recommendation?

| 1. Very difficult | 2. Somewhat difficult | 3. Somewhat easy | 4. Very easy |
| --- | --- | --- | --- |

*□ This is particularly important to my mental health, and I would like to discuss this further*

1. How satisfied are you with your communication with doctors (physical health care and/ or psychiatrists)?

| 1. Very satisfied | 2. Somewhat satisfied | 3. Somewhat dissatisfied | 4. Very dissatisfied |
| --- | --- | --- | --- |

*□ This is particularly important to my mental health, and I would like to discuss this further*

1. How do you experience accessing the medicines you need for you physical and/or mental health?

| 1. Very difficult | 2. Somewhat difficult | 3. Somewhat easy | 4. Very easy | 5. Not applicable |
| --- | --- | --- | --- | --- |

*□ This is particularly important to my mental health, and I would like to discuss this further*

1. From your point of view, how does our society perceive people who use mental health services?

| 1. Very negative | 2. Somewhat negative | 3. Neutral | 4. Positive |
| --- | --- | --- | --- |

*□ This is particularly important to my mental health, and I would like to discuss this further*

1. Generally, how do you experience accessing enough healthy food?

| 1. Very difficult | 2. Somewhat difficult | 3. Somewhat easy | 4.Very easy |
| --- | --- | --- | --- |

*□ This is particularly important to my mental health, and I would like to discuss this further*

1. Is there anything you want to say about the answers you have given to the questions in this block? Or, is there anything you would like to add about how your access to health care and food affect your mental health?

**Neighbourhood, transportation, housing**

1. How worried are you about safety in your neighbourhood?

| 1. Very worried | 2. Somewhat worried | 3. Hardly worried | 4. Not worried at all |
| --- | --- | --- | --- |

*□ This is particularly important to my mental health, and I would like to discuss this further*

1. How satisfied are you with your sense of belonging to your local neighbourhood community? A strong sense of belonging would mean that you feel valued by others and treated as an important part of the community.

| 1. Very satisfied | 2. Somewhat satisfied | 3. Somewhat dissatisfied | 4. Very dissatisfied |
| --- | --- | --- | --- |

*□ This is particularly important to my mental health, and I would like to discuss this further*

1. In the past 12 months, has lack of suitable transportation kept you from any of the following? Transportation includes here public (bus, train, etc.) and personal (car, bicycle etc.) means of transportation. (Please check **all** that apply)

| From medical appointments or getting medications |  |
| --- | --- |
| From non-medical appointments, meetings, work, or getting things that you needed |  |
| From social meetings, meeting friends, family etc. |  |
| From nothing |  |

| From something else |  |
| --- | --- |

*□ This is particularly important to my mental health, and I would like to discuss this further*

1. What is your housing situation today? (Please choose **one** of the following options)

| You do not have housing (Staying with others, in a hotel, in a shelter, in an abandoned building, or outside in public spaces). |  |
| --- | --- |
| You currently live in an institution and do not have private housing (living on a hospital, prison etc.) |  |
| You have housing today, but you are worried about losing housing in the future. |  |
| You have stable housing. |  |

*□ This is particularly important to my mental health, and I would like to discuss this further*

1. Which of the following best describes your living arrangements? (Please check **all** that apply)

| Live alone |  |
| --- | --- |
| Live with partner |  |
| Live with child(ren) |  |
| Live with parent(s)/other related adult(s) |  |
| Live with unrelated adult(s) |  |

*□ This is particularly important to my mental health, and I would like to discuss this further*

1. Thinking of your housing situation, are you exposed to any of the following problems? (Please check **all** that apply)

| Insects (e.g., cockroaches) or rodents |  |
| --- | --- |
| General dirtiness |  |
| Disrepair |  |
| Landlord disputes |  |
| Housing conditions that are incompatible with your health conditions |  |
| Mould or dampness |  |
| Threat of eviction |  |
| Overcrowding (more people living in the household than there are rooms) |  |
| Other |  |

*□ This is particularly important to my mental health, and I would like to discuss this further*

1. In the past 12 months, how often have you moved from one home to another?

| 1. Too frequently | 2. A few times | 3. Once | 4. Never |
| --- | --- | --- | --- |

*□ This is particularly important to my mental health, and I would like to discuss this further*

1. In the past 12 months, have you ever been forced to sleep outside, in a shelter, or in a place not meant for sleeping?

| Yes | No |
| --- | --- |

*□ This is particularly important to my mental health, and I would like to discuss this further*

1. Within the past 12 months, have you ever been unable to get one of the following when it was really needed? (Please check **all** that apply)

| Heat |  |
| --- | --- |
| Electricity |  |
| Water |  |
| Access to a working phone |  |
| Internet |  |

*□ This is particularly important to my mental health, and I would like to discuss this further*

1. Is there anything you want to say about the answers you have given to the questions in this block? Or, is there anything you would like to add about how your neighbourhood and housing affect your mental health?

**Social network, friends, family, and caring responsibility**

1. How much do you trust people in general?

| 1. A lot | 2. A fair amount | 3. Not very much | 4. Not at all |
| --- | --- | --- | --- |

*□ This is particularly important to my mental health, and I would like to discuss this further*

1. How satisfied are you with the number of social networks you are a member of? These could be formally organised social networks (like a political party or a sports team), or just groups of people who get together regularly to do an activity or talk about things (like a group of friends, or a carpool community).

| 1. Very satisfied | 2. Somewhat satisfied | 3. Somewhat dissatisfied | 4. Very dissatisfied |
| --- | --- | --- | --- |

*□ This is particularly important to my mental health, and I would like to discuss this further*

If member of any groups:

B.) How often do you attend meetings of the social networks or groups you belong to?

| 1. Frequently | 2. Sometimes | 3. Rarely | 4. Never |
| --- | --- | --- | --- |

*□ This is particularly important to my mental health, and I would like to discuss this further*

1. How satisfied are you with your relationships with the people that are close to you (relatives and/or friends)?

| 1. Very satisfied | 2. Somewhat satisfied | 3. Somewhat unsatisfied | 4. Very unsatisfied |
| --- | --- | --- | --- |

*□ This is particularly important to my mental health, and I would like to discuss this further*

1. How worried are you about being lonely or isolated from those around you?

| 1. Very worried | 2. Somewhat worried | 3. Hardly worried | 4.Not worried at all |
| --- | --- | --- | --- |

*□ This is particularly important to my mental health, and I would like to discuss this further*

1. Thinking of the support you receive from others around you, which of the following statements describe your situation? (Please check **all** that apply)

| *I experience a lot of understanding and comfort from others*. |  |
| --- | --- |
| *I know a very close person whose help I can always count on.* |  |
| *I know several people with whom I like to engage in things with.* |  |
| *If I am down, I know who I can go to.* |  |

*□ This is particularly important to my mental health, and I would like to discuss this further*

1. Thinking now specifically of your family, are you experiencing any of the following in your family? (Please check **all** that apply)

| Major problems in relationship with spouse/ partner |  |
| --- | --- |
| Major problems in relationship with parents or in-laws |  |
| Major problems in relationship with children |  |
| Absence of family member |  |
| Disappearance of family member(s) |  |
| Separation of family |  |
| Substance abuse by a family member |  |

*□ This is particularly important to my mental health, and I would like to discuss this further*

1. Do you have unpaid caring responsibilities for any of the following? (Please check **all** that apply)

| Partner |  |
| --- | --- |
| Child |  |
| Parent |  |
| Other family members |  |
| Friend |  |
| No caring responsibilities |  |

*□ This is particularly important to my mental health, and I would like to discuss this further*

If caring responsibilities for anyone

B.) How many hours in total do you usually spend on caring responsibilities?

| 0-5 hours a week | 5-20 hours a week | 20-40 hours a week | More than 40 hours a week |
| --- | --- | --- | --- |

*□ This is particularly important to my mental health, and I would like to discuss this further*

If caring responsibilities for child(ren)

C.) Who is responsible for taking care of your child(ren)? (Please check **all** that apply)

| You alone |  |
| --- | --- |
| You with partner (**biological or adoptive parent***)* |  |
| You with partner (**neither biologically nor legally** related to the child) |  |
| You with other family members of the child(ren) |  |
| You with representatives of the state |  |
| You with someone else, not listed in the answer options |  |

*□ This is particularly important to my mental health, and I would like to discuss this further*

D.) How do you experience, getting access to affordable and reliable child-care?

| 1. Very difficult | 2. Somewhat difficult | 3. Somewhat easy | 4.Very easy |
| --- | --- | --- | --- |

*□ This is particularly important to my mental health, and I would like to discuss this further*

1. If for any reason you need help with activities of daily living such as bathing yourself, preparing meals, shopping, managing finances, etc., how satisfied are you with the help you receive?

| 1. Very satisfied | 2. Somewhat satisfied | 3. Somewhat unsatisfied | 4.Very unsatisfied | 5. Not applicable |
| --- | --- | --- | --- | --- |

*□ This is particularly important to my mental health, and I would like to discuss this further*

1. Is there anything you want to say about the answers you have given to the questions in this block? Or, is there anything you would like to add about how your social network and your caring responsibilities affect your mental health?

**Adverse experiences in childhood and adulthood**

1. Thinking of your childhood (0-18 years), have you experienced any of the following? (Please check **all** that apply)

| Inadequate parental supervision |  |
| --- | --- |
| Upbringing away from parents in welfare custody/ in care |  |
| Upbringing away from parents in institutions, such as boarding school |  |

*□ This is particularly important to my mental health, and I would like to discuss this further*

1. Thinking of your childhood, have you experienced any of the following acts of violence? (Please check **all** that apply)

|  | By family member | By someone outside the family |
| --- | --- | --- |
| Witnessing domestic violence, without being the victim of the violence yourself |  |  |
| Being the victim of physical abuse (repeated attacks with implements, beatings) |  |  |
| Being the victim of sexual abuse (sexual act, sexual contact, or exploitation) |  |  |
| Being the victim of psychological abuse (humiliation, terrorising, deprivation of basic needs, extreme rejections, corruption, or blackmail) |  |  |
| Being the victim of bullying (an ongoing and deliberate misuse of power in relationships through repeated verbal, physical and/or social behaviour that intends to cause harm) |  |  |

*□ This is particularly important to my mental health, and I would like to discuss this further*

1. Have you been subject to any of the following adverse events and conditions, in childhood or adulthood? (Please check **all** that apply)

| Natural disaster (such as hurricane, flood, earthquake, tornado etc.) with a threat to life |  |
| --- | --- |
| War |  |
| Violent conflicts (such as riots, strikes etc.) |  |
| Acts of terrorism |  |
| Witnessing a severe trauma to a loved one |  |
| Lack of learning and play experience |  |
| Unwanted pregnancy as (potential) parent |  |
| Hostility towards and scapegoating of you |  |
| Loss of a significant relationship (death or permanent departure of sibling, parent, partner, very close friend) |  |

*□ This is particularly important to my mental health, and I would like to discuss this further*

1. Have you ever been held against your will?

| 1. For several *years* | 2. For several *months* | 3. For several *hours*/*days* | 4. Never |
| --- | --- | --- | --- |

*□ This is particularly important to my mental health, and I would like to discuss this further*

*If held against the will before:*

B.) Please specify the circumstances in which you have been held

| Sectioned under the mental health act |  |
| --- | --- |
| In prison |  |
| In immigrant detention facilities |  |
| Illegal abduction |  |
| Other |  |

*□ This is particularly important to my mental health, and I would like to discuss this further*

1. How worried are you about your physical, emotional, and sexual safety in your home?

| 1. Very worried | 2. Somewhat worried | 3. Hardly worried | 4. Not worried at all |
| --- | --- | --- | --- |

*□ This is particularly important to my mental health, and I would like to discuss this further*

1. Within the past year, how often did anyone, including family, physically hurt you?

| 1. Frequently | 2. Sometimes | 3. Rarely | 4. Never |
| --- | --- | --- | --- |

*□ This is particularly important to my mental health, and I would like to discuss this further*

1. Within the past year, how often did anyone, including family, insult or talk down to you?

| 1. Frequently | 2. Sometimes | 3. Rarely | 4. Never |
| --- | --- | --- | --- |

*□ This is particularly important to my mental health, and I would like to discuss this further*

1. Within the past year, how often did anyone, including family, try to control your spending or access to cash, assets and finances?

| 1. Frequently | 2. Sometimes | 3. Rarely | 4. Never |
| --- | --- | --- | --- |

*□ This is particularly important to my mental health, and I would like to discuss this further*

1. Have you ever been raped or forced to have any kind of sexual activity? That is sexual contact that either at the time or looking back on it now was unwanted.

| Yes | No |
| --- | --- |

*□ This is particularly important to my mental health, and I would like to discuss this further*

1. Is there anything you want to say about the answers you have given to the questions in this block? Or, is there anything you would like to add about how your adverse experiences in childhood and adulthood affect your mental health?

**Losses, life events, and physical health**

1. Many people loose things that are valuable to them but do not have any monetary value. Within the past 12 months, have you lost something immaterial that was of great value to you? (Please check **all** that apply)

| I lost a beloved social role, defined as occupying a certain position or performing a particular function in society, like being a firefighter, or a parent etc. |  |
| --- | --- |
| I lost my social status, defined as prestige, respect, and esteem in the eyes of others. |  |
| I lost my autonomy or independence, defined as the ability to make your own decisions about your life. |  |
| No, I did not loose anything of this kind within the last 12 months. |  |

*□ This is particularly important to my mental health, and I would like to discuss this further*

1. Within the past 12 months, have you experienced any of the following turning points as stressful? (Please check **all** that applies:)

| Moving into your own accommodation |  |
| --- | --- |
| Starting a romantic relationship |  |
| Getting married |  |
| Becoming a parent |  |
| Breakdown of a romantic relationship |  |
| Children moving out |  |
| Retirement |  |

*□ This is particularly important to my mental health, and I would like to discuss this further*

1. In general, how satisfied are you with your physical health?

| 1. Very satisfied | 2. Somewhat satisfied | 3. Somewhat unsatisfied | 4. Very unsatisfied |
| --- | --- | --- | --- |

*□ This is particularly important to my mental health, and I would like to discuss this further*

1. Within the past month, how often did any problems with your (physical and mental) health interfere with your daily activities?

| 1. Frequently | 2. Sometimes | 3. Rarely | 4. Never |
| --- | --- | --- | --- |

*□ This is particularly important to my mental health, and I would like to discuss this further*

1. Is there anything you want to say about the answers you have given to the questions in this block? Or, is there anything you would like to add about how your losses, life events, and physical health affect your mental health?
